# Low intake of iodized salt and iodine containing supplements among pregnant women with apparently insufficient iodine status - time to change policy?

**DOI:** 10.1186/s13584-020-00367-4

**Published:** 2020-03-30

**Authors:** Shani R. Rosen, Yaniv S. Ovadia, Eyal Y. Anteby, Shlomo Fytlovich, Dorit Aharoni, Doron Zamir, Dov Gefel, Simon Shenhav

**Affiliations:** 1 School of Nutritional Science; Institute of Biochemistry, Food Science and Nutrition; Robert H. Smith Faculty of Agriculture, Food and Environment, The Hebrew University of Jerusalem, 76100 Rehovot, Israel; 2 Obstetrics and Gynecology Department, “Barzilai” University Medical Center Ashkelon, Ashkelon, Israel; 3 Foreign studies department; Robert H. Smith Faculty of Agriculture, Food and Environment, The Hebrew University of Jerusalem, Jerusalem, Israel; 4 Faculty of Health Sciences, Ben-Gurion University of Negev, Ashkelon, Israel; 5 Laboratory of Clinical Biochemistry, Barzilai University Medical Center Ashkelon, Ashkelon, Israel; 6 Internal Medicine Department, Barzilai University Medical Center Ashkelon, Ashkelon, Israel

**Keywords:** Thyroglobulin, Iodine, Desalination, Pregnancy, Thyroid, Nutrition

## Abstract

**Background:**

Iodine is an essential nutrient for human health throughout the life cycle, especially during early stages of intrauterine life and infancy, to ensure adequate neurocognitive development. The growing global reliance on desalinated iodine-diluted water raises the specter of increased iodine deficiency in several regions. The case of Israel may be instructive for exploring the link between iodine status and habitual iodine intake in the setting of extensive national reliance on desalinated water. The aim of this study was to explore the relationship between iodine intake, including iodized salt and iodine-containing supplements intake, and iodine status among pregnant women residing in a sub-district of Israel that is highly reliant on desalinated iodine-diluted water.

**Methods:**

A total of 134 consecutive pregnant women were recruited on a voluntary basis from the obstetrics department of the Barzilai University Medical Center during 2018. Blood was drawn from participants to determine levels of serum thyrotropin (TSH), thyroid peroxidase antibodies (TPOAb), thyroglobulin antibodies (TgAb) and thyroglobulin (Tg). An iodine food frequency questionnaire (sIFFQ) was used to assess iodine intake from food, IS and ICS. A questionnaire was used to collect data on demographic and health characteristics.

**Results:**

A total of 105 pregnant women without known or reported thyroid disease were included in the study. Elevated Tg values (≥ 13 μg/L), were found among 67% of participants, indicating insufficient iodine status. The estimated iodine intake (median, mean ± SD 189, 187 ± 106 μg/d by sIFFQ) was lower than the levels recommended by the World Health Organization and the Institute of Medicine (250 vs. 220 μg/day respectively). The prevalence of iodized salt intake and iodine containing supplement intake were 4 and 52% (respectively). Values of Tg > 13 μg/L were inversely associated with compliance with World Health Organization and Institute of Medicine recommendations.

**Conclusions:**

While the Israeli Ministry of Health has recommended the intake of iodized salt and iodine containing supplements, this is apparently insufficient for achieving optimal iodine status among Israeli pregnant women. The evidence of highly prevalent probable iodine deficiency in a sample of pregnant women suggests an urgent need for a national policy of iodized salt regulation, as well as guidelines to promote iodine containing supplements and adherence to them by caregivers. In addition, studies similar to this one should be undertaken in additional countries reliant on desalinated iodine-diluted water to further assess the impact of desalinization on maternal iodine status.

## Background

Decades of public health research have established that iodine deficiency (ID) in pregnancy may impair neurological development of the offspring. Iodine is a crucial element for brain evolvement, especially during pregnancy when fetal brain development is very rapid. Notably, ID may result in compromised perinatal outcomes such as stillbirth, pre-eclampsia and cretinism. Additionally, ID is associated with compromised outcomes throughout life, such as altered IQ and cognition levels [1, 2]. Globally, ID is the leading cause of preventable intellectual deficits [3]. In iodine sufficient areas, pregnant women (PW) may maintain stable total body iodine levels throughout pregnancy. However, in mild to moderate iodine deficient areas, total body iodine stores often decline throughout pregnancy. Moreover, iodine intake has a key role in thyroid function during pregnancy even in iodine sufficient areas [3, 4].

Inadequate iodine status is common among PW worldwide [5–7]. Out of 72 countries with data on iodine status based on median urinary iodine concentration (mUIC), approximately 54% are classified as ID, including countries in developed regions [5, 8]. The Israel National Iodine Survey (INIS) conducted in 2016, has shown that Israel is an ID country. Mild ID was found among a national representative sample of school-aged children (*n* = 1023) and substantial iodine insufficiency was found in a representative sample of PW (*n* = 1074) [9]. Of PW spot UIC samples, 85% were below the World Health Organization (WHO) adequacy range (150–249 μg/L [10, 11]), and the mUIC was 61 μg/L. These data have placed Israel in the worst decile globally for iodine status [6]. The prevalence and degree of ID that was found in the INIS suggests that the population may not be able to fulfill its intellectual potential [6, 9]. Thus, the ID found among PW indicates a serious public health concern in Israel.

In the wake of the INIS findings, the Israeli Ministry of Health (MOH) promoted an iodine policy in early 2017, recommending replacement of regular table salt with iodized salt (IS), without increasing the amount of salt intake [12, 13]. In late 2017, the MOH recommended intake of iodine containing supplements (ICS) with 150–250 μg/d iodine, starting 1 month prior to a planned pregnancy and continuing throughout lactation [12, 14, 15]. However, a mandatory national salt iodization policy has not yet been established and the MOH has only recommended IS intake on a voluntary basis [9, 13, 16]. Regarding ICS, the MOH recommendations have not been accompanied by specific and clear guidelines to promote relevant adherence by health care professionals [9, 12, 14].

The reasons for the substantial ID found in Israeli PW are not fully understood. Recent data from the INIS suggest that national ID may be attributed, in part, to substantial reliance on iodine-diluted desalinated water (DIDW) [7, 9]. The INIS researchers also noted that IS availability and historical reporting of ICS intake was low [9]. However, the INIS was limited by the unavailability of medical records and detailed information regarding iodine intake. Therefore, information is needed on the local association between the iodine status of PW and habitual dietary iodine. Such information is important for policy makers in Israel and elsewhere, in order to develop an efficient and relevant iodine policy. Moreover, in light of the prevalence of ID among PW in both developing and developed regions around the world, exploring this link can contribute to the development of more definitive polices in Israel and globally.

The growing prevalence of water desalination globally [17] highlights the importance of data regarding the impact of habitual dietary iodine intake on iodine status [7, 17, 18]. Such information may be crucial in areas with DIDW reliance, which may diminish iodine intake [7, 19]. Israel relies extensively on DIDW [7, 20]. The case of Israel’s Ashkelon sub-district can be instructive, because stable estimates of DIDW exposure were available for this sub-district [19].

Accordingly, the aim of this study was to explore the relationship between iodine status and iodine-related dietary habits (including IS use and ICS intake), among PW from an area of Israel that is highly reliant on DIDW.

## Methods

### Participants, settings and design

**Fig. 1:**
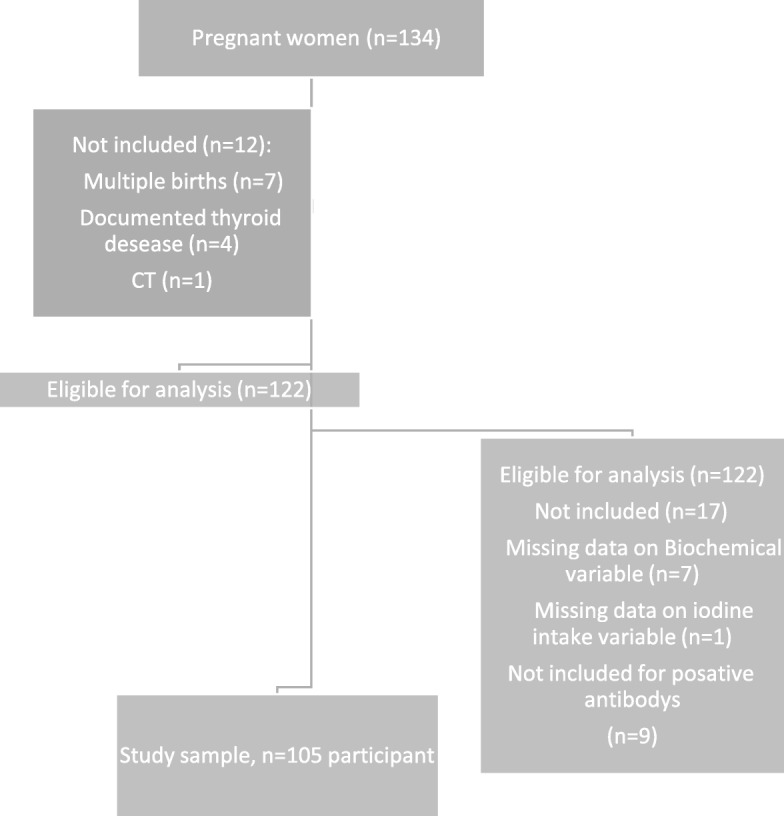
Flow chart describing the screening process and the study sample generation. CT = Computed tomography scan

The research proposal was approved by the Helsinki Committee at The Barzilai University Medical Center in Ashkelon (BUMCA). All PW who participated in this study provided written informed consent after the research protocols were explained in detail (Fig. 1).

**Fig. 2:**
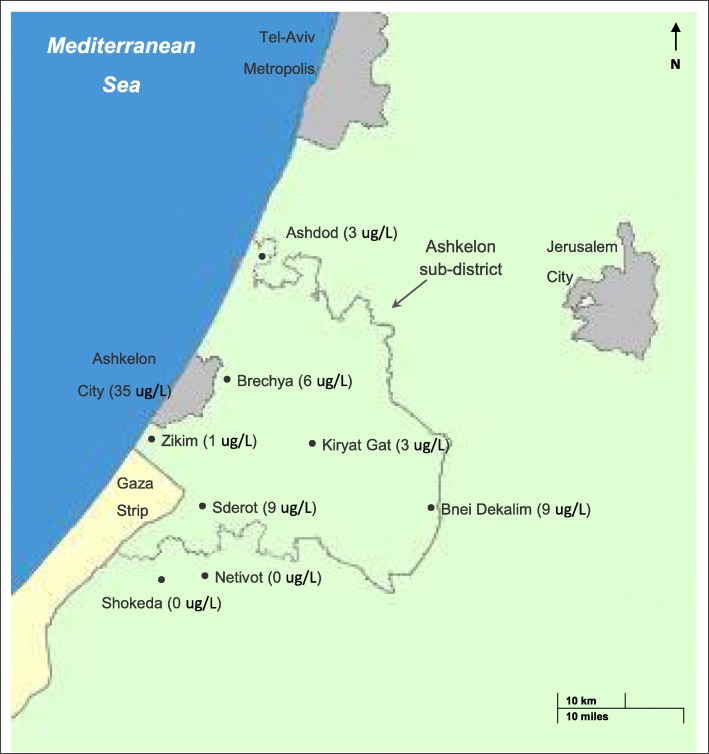
Map of Israel's southern coastal area, showing Ashkelon sub-district and its surroundings. * Municipalities in which participants reported drinking unfiltered tap water are presented in bullet point and the estimated annual average of unfiltered tap water iodine concentrations (in ug/L) are shown in parentheses after the name of the locality: Ashkelon City (35 μg/L), Ashdod (3 μg/L), Kiryat Gat (3 μg/L), Sderot (9 μg/L), Bnei Dekalim (9 μg/L), Brechya (6 μg/L), Netivot (0 μg/L), Zikim (1 μg/L), Shokeda (0 μg/L)

The study took place at the Obstetrics and Gynecology Department of BUMCA. The BUMCA is located in the Ashkelon sub-district, a geographical area with extensive reliance on DIDW since 2011 [21]. Figure 2 presents a map showing the location of the city of Ashkelon (where the BUMCA is located), and the Ashkelon sub-district location and area.

The study was a cross-sectional study. Consecutive PW were prospectively enrolled to the study between May and December of 2018. Participants provided blood samples and responded to the study questionnaires, along with written informed consent. The exclusion criteria were: multiple pregnancies, intake of medications that interfere with thyroid function (e.g., amiodarone, glucocorticoids, lithium-containing), previously reported or documented TD, and any indications of an underlining TD (such as TSH > 10 mIU/L, and positive thyroid antibodies: Thyroglobulin antibody (TgAb) values above 40 IU/ ml and thyroid peroxidase (TPOAb); values above 35 IU/ml were considered positive as considered by previous studies [22]). Cases with positive TgAb values were not included because TgAb can interfere with serum thyroglobulin (Tg) analysis [23]. We considered iodine status to be sufficient (Tg ≤ 13 μg/L) or insufficient (Tg >13 μg/L) according to a recently suggested standard [22].

### Assays

The Tg level was used as a biomarker of iodine status [23, 24]. Blood samples were used for data on the levels of TSH, thyroid antibodies, TPO-ab, Tg-ab, Tg, free triiodothyronine (FT3), and free thyroxine (FT4), which were analyzed at the clinical biochemistry laboratory of the BUMCA, using electrochemiluminescence immunoassay on Modular Analytics E601 analyzer (Cobas, Roche Diagnostics GmbH, Mannheim, Germany). Reference ranges were 0.27–4.2 mU/L for TSH, 3.1–6.3 pmol/L for FT3, and 0.93–1.7 ng/dL for FT4.

### Iodine intake assessment using semi-quantitative iodine food frequency questionnaire

A previously used, semi-quantitative iodine food frequency questionnaire (sIFFQ) with a focus on iodine rich foods, designed for the Israeli adult population was used in order to assess habitual iodine intake as reported elsewhere [18]. Estimates of the iodine content of the foods consumed by study participants were derived from multiple sources: specific food items from the Department of Nutrition at the MOH (iodine composition investigation in food by Dr. Eli Havivi 1989); fresh water fish from the Agricultural Service of Israel and the Israeli Fish Breeders Association (fresh water fish nutritional composition, 2012) [21, 25]. In brief, iodine intake was estimated for foods of marine origin (including fish and sea food), milk products (including milk, cheese and yogurt), water, and other significant sources of iodine [21]. 

In order to quantify the iodine intake from water, we used a quantitative model, which was previously used in Israel [19]. In this model, iodine from locally retailed bottled drinking water were considered negligible due to low content of iodine (0–10 μg/L [26]). Filtered water was also excluded due to the high iodine variability of filtered water (in the range of 0–27 μg/L [19]). To model the contribution of drinking-water to daily iodine intake, we used geographic locality specific estimates of water iodide concentration. Content of iodine from water was calculated using geographic locality specific estimates of water iodide concentration. Mean iodide concentrations were estimated using data published by the MOH and the estimated annual proportion of DIDW supply reported by Mekorot Israel National Water Company for specific municipalities in the Ashkelon district (personal communication, Mr. Yuri Kasperuk, Israel’s Southern Region Water Supply Engineer at Mekorot Israel National Water Co.). The extent of iodine intake via ICS was calculated using data from the manufacturers regarding the intake of the ICS, and self- reported detailed information from the study questionnaire regarding type of ICS, and the frequency and duration of ICS use.

### Additional data sources for variables related to iodine status

Possible correlates of iodine status were examined using information obtained from medical records (regarding general medical background) as well as from the patient survey (regarding patient demographics, health, a history of thyroid disease, and weight gain during gestation). Correlation between biomarkers and the sIFFQ was used to provide better assessment regarding iodine status, as they are complementary measurements: Tg reflects Iodine intake of weeks to months, and sIFFQ reflects iodine intake during the previous year [19, 23]. Spot urinary iodine concentration (UIC) is a common measure in population studies [27]. However, it was not considered suitable for this study, because of three reasons: (1) the high within-individual variability in UIC [28], (2) UIC indicates recent iodine intake (days), whereas MOH recommend achieving adequate iodine intake at least 1 month prior to conception and the study sample consists entirely of PW [12, 29], (3) The sample size of our study (*N* = 105) was not large enough (*N* ≥ 500) to level out the day-today variation in iodine intake and urinary volume [19, 21].

### Statistical analysis

Eligible data and samples were analyzed using IMB SPSS statistics software (version 25.0 Chicago IL USA). The Tg values were dichotomized to ≤13 μg/L Tg >13 μg/L (sufficient vs. insufficient, respectively). Possible correlates of sufficient iodine status were examined using multiple logistic regression. Results were analyzed by pregnancy trimester, ICS intake, and locality. Maternal education level, smoking status, and iodine intake during gestation were included in the analysis. Correlation between biomarkers and the sIFFQ was analyzed using a Pearson correlation coefficient. Results are described as mean ± SD. A two tailed *P*-value < 0.05 was considered statistically significant.

## Results

### Participants

Of the 134 consecutive PW who originally enrolled in the study, 29 women were not included because they did not meet study eligibility criteria. Ultimately, 105 women were included in the analysis. Exclusions were due to multiple pregnancies (*n* = 7), documented thyroid disease (*n* = 4), Computed tomography scanning (*n* = 1), missing data on biochemical and questioner variables (*n* = 8) and thyroid antibody positivity (*n* = 9). This screening process and the study sample generation is described in Fig. 1.

Characteristics of participants are presented in Table 1. Mean ± SD age was 31 ± 7 years, mean pre-conception BMI 25 ± 6 kg/m^2^. 18% of the participants were over age 35 and 76% were in their third trimester. 17% of participants were smokers at study entry, 46% had graduated college, 72% were born in Israel, and 45% had two or more children.

**Table 1:** Socio-demographic characteristics of the study sample

	Number	Percent	Mean	Median
Total *n* = 105				
Maternal age (years)	**95**	**100**	**31 ± 5**	**31**
Up to 25	15	14		
26–35	71	75		
Over 35	19	18		
Gestational age at study entry, weeks	**95**	**100**	**31 ± 7**	**32**
First trimester	4	4		
Second trimester	19	20		
Third trimester	72	76		
Smoking status	**105**	**NA**		
Smoked before pregnancy	34	34		
Smoking at study entry	18	17		
No smoking	66	66		
Education	**105**	**100**	NA	NA
Graduated college	48	46		
Other	57	54		
Country of birth	**105**	**100**	NA	NA
Israel	72	72		
Other	28	28		
Number of children	**100**	**100**	**1.58 ± 1.57**	**1**
None	28	29		
One	27	28		
Two or more	45	45		
Preconception BMI, kg/m2	**63**	**100**	**25 ± 6**	**24**
Underweight (less than 18.5)	4	6		
Normal weight (18.5–24.9)	31	49		
Overweight (24.9–29.9)	20	32		
Obese (30–34.9)	5	8		
Extremely obese (35 and higher)	3	5		

*NA* not available, *BMI* body mass index

### Maternal iodine status assessment by Tg based on blood samples

The median concentration of Tg cross-trimester was 17 μg/L, (mean ± SD 25 ± 27 μg/L), and 14% of Tg values were less than or equal to 40 μg/L. Median and mean ± SD concentrations were, TSH (*n* = 104, 1.6, 1.6 ± 0.8 μIU/mL), FT3 (*n* = 102, 4.2, 4.2 ± 0.6 pmol/L) and FT4 (*n* = 101, 1, 1 ± 0.1 ng/dL). Iodine status sufficiency was assessed using two cut-off values of Tg. Of the participants, 67% did not meet the Tg ≤13 μg/L standard, while 14% did not meet the Tg > 40 μg/L standard. Median Tg was 17 μg/L. These parameters did not differ significantly by week of pregnancy, intake of goitrogenic foods, or smoking status (data not presented). They did vary significantly by iodine intake for Tg ≤13 μg/L and for Tg > 40 μg/L (*p*<0.05, *p*<0.001, respectively) and by ICS intake for Tg > 40 μg/L (*p*<0.001).

### Habitual dietary iodine intake assessment by sIFFQ

**Fig. 3:**
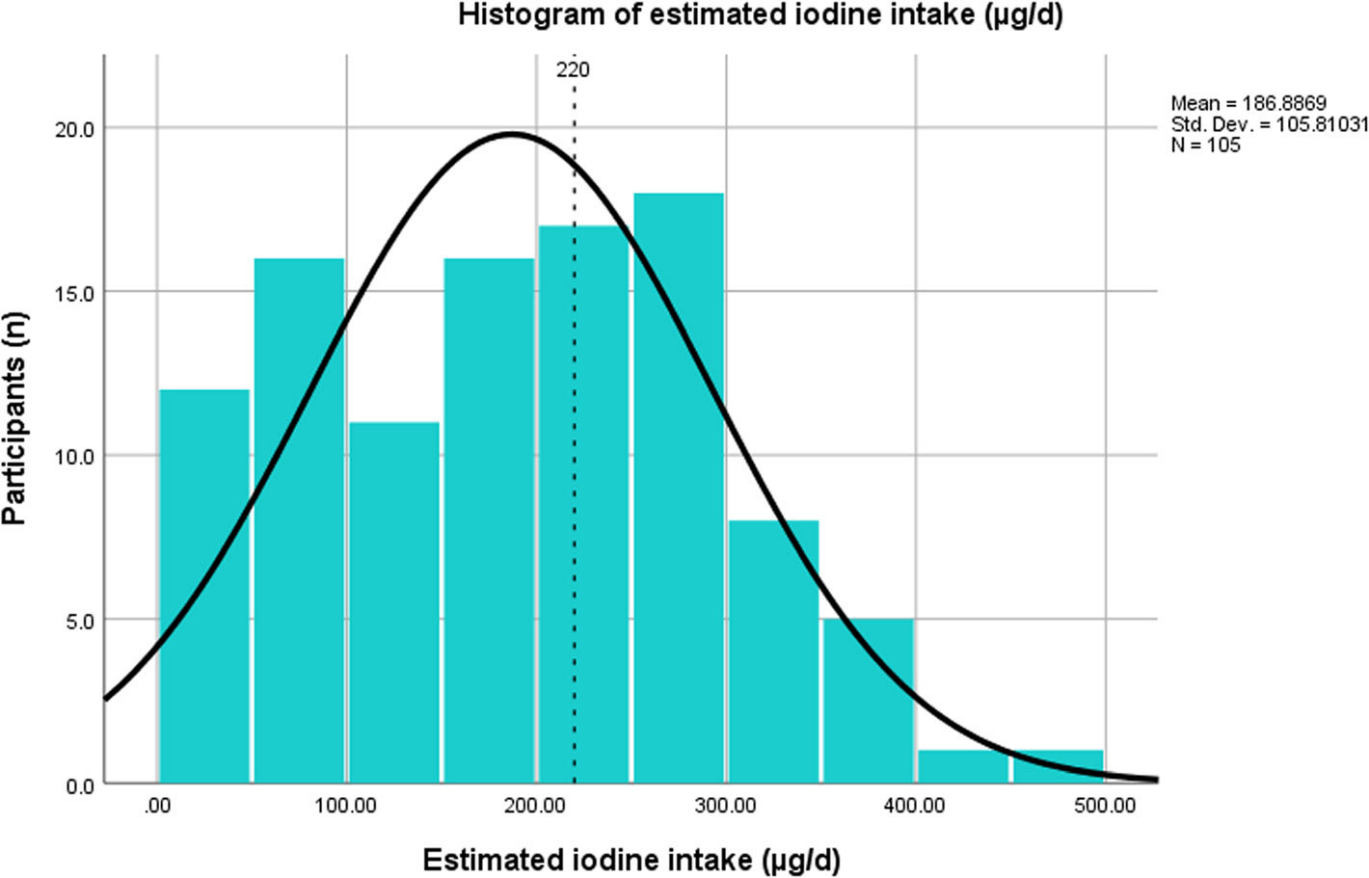
Distribution of estimated daily iodine intake (μg/d) of the study sample according to sIFFQ (*n* = 105). Note: Reference line represents IOM and DRI guidelines for iodine intake (220 μg/d) from food, IS and ICS. μg/d = micrograms per day; sIFFQ = semi-quantitative iodine food frequency questionnaire; IOM = Institute of Medicine; DRI = dietary reference intake; IS = iodized salt; ICS = iodine-containing supplements; WHO=World Health Organization

The median iodine intake from food and ICS was 189 μg/d (mean 187 ± 106 μg/d), whereas the median iodine intake from food alone was 80 μg/d (mean 96 ± 64 μg/d) and from food and water was 94 μg/d (mean 106 ± 66 μg/d). Full distribution of the study sample’s estimated daily iodine intake (μg/d) according to sIFFQ (*n* = 105) is presented in Fig. 3. Intake of IS was reported by 4% of participants (*n* = 4). The proportion of participants reporting intake of ICS during gestation was 52% (*n* = 55), mostly starting intake by the end of the first trimester (mean week 8 of gestation, median week 8 of gestation). The proportion of participants that reported intake of ICS prior to pregnancy was 11% (*n* = 80). Almost half (48%) of the participants reported intake of unfiltered tap water on a daily basis, with a mean value of 0.5 l/ day. Notably, 14% of PW reported intake of at least 1 liter/day. Among participants who reported drinking tap water, the average contribution of tap water to the daily mean iodine intake was 19 μg/d - 9% of the Institute Of Medicine (IOM) recommendation for daily adequate iodine intake - 220 ug [30]. Annual average iodine concentrations (in ug/L) of unfiltered tap water are shown in Fig. 2 for localities which had respondents who reported drinking unfiltered tap water.

As indicated in Table 2, in total, 41% of participants complied with IOM iodine intake recommendations. Compliance with recommendations was associated with a report of ICS intake (*p*<0.001). Among the participants that reported intake of ICS, 71% complied with recommendations. Among the participants that reported no intake of ICS, 8% complied with recommendations. In total, 31% of participants complied with WHO iodine intake recommendations. Compliance with recommendations was associated with a report of ICS intake (*p*<0.001). Among the participants that reported intake of ICS, 55% complied with the recommendations of IOM. Of the participants who did not report ICS intake, 6% complied with IOM recommendations [30]. The difference between the groups is statistically significant (*p*<0.001).

**Table 2:** Percent of PW meeting iodine intake standards stratified by the taking of supplements

	N	≥220 μg iodine/day (IOM standard)	≥250 μg iodine/day (WHO standard)
Total	**105**	**41%**	**31%**
Taking supplements containing iodine	55	71%	55%
Not taking supplements containing iodine	50	8%	6%
*P* value		.00	.00

*IOM* [11, 30]

*PW* Pregnant Women, *IOM* Institiue of Medicien, *WHO* World Health Organization

**Fig. 4:**
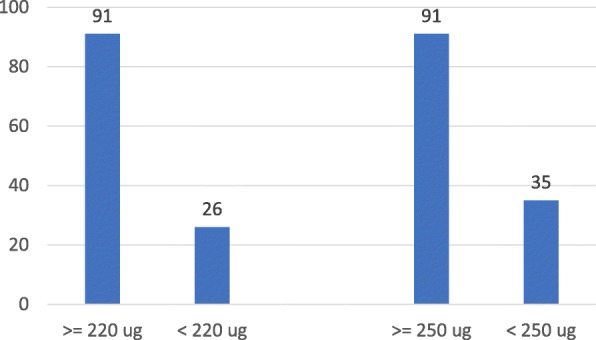
Percent of PW reporting ICS among the study sample, stratified by level of iodine intake (as assessed by sIFFQ). PW = Pregnant women; sIFFQ = semi-quantitative iodine food frequency questionnaire

As indicated in Fig. 4, among participants who complied with IOM iodine intake recommendations [30], 91% reported ICS intake. The proportion of reported ICS intake was 26% among participants who did not meet the IOM recommendation [30] (*p*<0.001). These proportions were 91% and 35% (respectively) when compared to the WHO standard [11] (*p*<0.001).

### The relationship between iodine status and iodine intake

As indicated in Table 3, Tg concentrations were correlated with intake of iodine from food and ICS. In total, 67% of participants had a Tg value of over 13 μg/L. This figure was 74% among participants who did not meet the IOM standard for iodine intake (220 μg/d) [30], and 56% among participants who did meet the IOM standard. The difference between the groups is statistically significant (*p*<0.05).

**Table 3:** The relationship between Tg cut-off values and iodine intake in the study sample

	N	Tg > 13 μg/d	Tg > 40 μg/d
Iodine intake	105		
≥ 220 μg/d	43	56%	5%
< 220 μg/d	62	74%	21%
*P* value		.049	.019
Intake of iodine containing supplements			
Yes	55	60%	4%
No	50	74%	26%
*P* value		0.12	0.001

IOM standard for iodine intake (220 μg/d, [30]), includes intake from both food and supplements

*Tg* Serum thyroglobulin, *IOM* Institute Of Medicine

In total, 14% of participants had a Tg value of over 40 μg/L. This figure was 21% among participants who did not meet the IOM standard for iodine intake (220 μg/d) [30], and 5% among participants who complied with IOM recommendations. The difference between the groups is statistically significant (*p*<0.05).

## Discussion

### Summary of key findings

Our study found that 14% of the participants had a Tg value of over 40 μg/L. This can be compared with a proposed standard which suggests that a population is iodine sufficient only if less than 3% of the population has Tg values greater than 40 μg/L(proposed indicator for population iodine status adequacy) [22, 31]. It is also noteworthy that 67% of the participants in the study sample had a Tg value of over 13 μg/L, as another proposed standard is that a population is iodine sufficient only if the median Tg is less than 13 μg/L [22, 31]. As for iodine intake, 41% of participants met the IOM standard for iodine intake [30]. In our sample, a Tg level over 40 μg/L was inversely associated with meeting IOM iodine intake recommendations (*p*<0.05) and with intake of ICS (*p*<0.001). Furthermore, a Tg level of over 13 μg/L was inversely associated with achieving IOM iodine intake recommendations (*p*<0.05).

The study also found that the percent of participants who reported intake of IS during gestation was 4%, similar to the low rate of 3% of locally retailed IS (almost all of which is produced in Israel [16]). Most locally retailed IS contains approximately 30 mg/Kg., so that intake of a teaspoon (5 g) of IS each day is sufficient for the general population. However, a teaspoon a day would supply only 68% of the Dietary Reference Intakes (DRI) for PW (DRI, IOM: 220 mg/day [12, 16, 32]). Thus, serious consideration needs to be given to ways to increase the availability and intake of IS, particularly in regions around the globe that are reliant on DIDW.

The Israeli MOH published updated guidelines in 2017 recommending intake of 150–250,150–250 μg/d of iodine, starting at least 1 month prior to a planned pregnancy. This study found, however, that only 52% of participants reported intake of ICS during gestation. Moreover, initiation of ICS intake was on average at week 8 of gestation, with only 11% of participants reporting initiation of ICS prior to pregnancy. Thus, this study found low adherence to the ICS intake recommended by the MOH. The low prevalence of ICS intake and the late time of initiation may be due in part to a lag in updating the Israeli gynecology guidelines with specific and clear new guidance on ICS. The MOH should encourage the gynecology profession to promptly update the profession’s nutrition guidelines. In addition, the MOH should communicate the importance of adequate iodine intake during pregnancy to the general public, the health plans, primary care physicians, and other health professionals.

### Unique aspects of this study

This is the first study of the iodine status among a sample of Israeli PW residing in an area with extensive reliance on DIDW, using Tg as an iodine probable status indicator. There is no well-established biomarker for measuring iodine status in an individual, but Tg may be a functional biomarker of iodine status in mild-moderate ID populations [23, 27]. Mild-moderate ID during pregnancy stresses the thyroid gland. An increase in Tg during pregnancy suggests that thyroid gland volume is increasing accordingly [23]. Tg is considered a more sensitive indicator of iodine repletion than TSH since it declines more rapidly with iodine repletion [23]. The Tg and the sIFFQ are complementary assessment methods of iodine status [23], therefore in this study we also used the sIFFQ method, which has been established as an accurate method for assessing long term usual intakes of foods as well as placing participants into levels of intake. The sIFFQ used in this study is estimated to capture 94–97% of the iodine intake [21].

### The desalination context

The growing shortage of fresh water has become a global problem [17, 18]. As part of the solution, sea water is being desalinated in an increasing number of countries, supplying approximately 118 cubic meters a day for more than 300 million people globally [7, 17, 18]. Israel is a pioneer in seawater desalination, where 80% of the drinking water comes from DIDW (based on accumulated annual reported proportion [7, 19, 20]).

Desalination is an important means to address the global water scarcity [18]. However, it can inadvertently present a new challenge in regions where drinking water has provided a substantial portion of the iodine needed to achieve the recommended dietary allowance (RDA) for iodine [7, 19]. Reliance on DIDW places PW at an increased risk for ID [7, 9]. Accordingly, knowledge regarding the iodine status of PW, and factors influencing that status, is important for designing intervention to address this problem – both in Israel and globally [7].

### The relationship between this study and earlier Israeli studies

Along with findings of geographic variation in iodine concentrations of local water sources, ID has been reported in Israel for decades [9, 33, 34]. As reported by Rosenthal et al., changes over time in the source of water, and its iodine content, have had significant impact on goiter incidence in the northern part of Israel [33]. Our findings are consistent with, and may help explain, the findings of the INIS regarding low iodine status among PW [7, 9]. In the INIS, spot urine samples of 1074 PW were collected and mUIC was examined by regions, religious sectors, and pregnancy trimesters. The INIS was the first national study to reveal that ID is a serious national public-health concern, with 85% of PW UIC samples resulting in values below the adequacy range (150–249 μg/l), and a mUIC of 61 μg/l (interquartile range (IQR) 36–97 l μg/d). Furthermore, a recent report from 2016, (*N* = 50), where most of the sample consisted of apparently healthy childbearing aged women from the Ashkelon sub-district, showed low iodine status, based on the high median Tg (21 ng/mL), and prevalent elevated Tg values (Tg ≥ 10 ng/mL, 76%). The study also found a median iodine intake below the recommended daily intake (RDA) (99 vs 150 μg/day respectively [35]).

The researchers who conducted the INIS indicated several reasons for insufficient iodine status, such as, reliance on IDDW, low IS availability and historical low reporting of ICS intake [9]. Our study suggests that the findings found in previous studies in Israel [7, 9] may indeed be due in part to the reasons hypothesized in the INIS, with low levels of ICS and IS intake. The iodine status assessment methods used in our study complement those used in the INIS. The INIS study used mUIC and our study used Tg and sIFFQ. An important strength of the INIS is that it is national in scope. Complementary strengths of our study are the availability of medical records and habitual dietary iodine intake information.

### Relevant studies from other countries

A review from 2014 examined the Tg values of iodine deficient PW, and found that the majority of studies reported median Tg values exceeding 13 μg/L [22]. A recent study from the UK (*n* = 230) found median Tg values of 21, 19, 23 μg/d in the first, second and third trimesters respectively [23]. Furthermore, 18% of PW had Tg values greater than 40 μg/L, well exceeding the standard of 3% proposed by Zimmerman et al. [31]. The Tg values were higher in the iodine deficient group, where ID was classified based on a UIC level of less than 150 μg/L. No association was found between TSH values and ID. Iodine intake from milk (as estimated by a food frequency questionnaire) was inversely associated with Tg values [23]. A study from 2018 by Mioto et al. conducted in Brazil (*n* = 273) examined PW from an iodine sufficient area. Those PW had a median Tg of 11.2 μg/L and only 3.3% had a value exceeding 40 μg/L; mUIC was 140 μg/L [4]. The extent of iodine insufficiency found in our study (a median Tg concentration of 17 μg/L and 14% exceeding 40 μg/L) was more moderate than what was found in the 2014 review, and more serious than what was found in the Mioto et al. study.

### Study limitations

Our study had several limitations. First, the cross-sectional design of the study limits our knowledge on the intra-individual thyroid function changes throughout pregnancy as well as in concordance with iodine availability. Second, the sIFFQ method is limited in its capacity to provide an exact estimation of food intake, although it is accurate for assessing long term usual intakes of foods as well as placing participants into levels of intake. Third, the sample size of this study was relatively small, thereby limiting the extent and reliability of sub-group comparisons. Furthermore, the generalizability of the study’s findings is limited by the lack of a sample from a site less dependent on desalination. Finally, the connivance sample consisted of volunteers attending the Obstetrics and Gynecology department of BUMCA; therefore the sample might not reflect the overall PW population in the Ashkelon sub-district. To limit the potential bias, we did not included PW with health conditions known to affect iodine status, such as thyroid dysfunction and PW who used medications that are known to effect iodine status or thyroid function.

### Avenues for further research

Our study showed prevalent iodine insufficiency (67% of Tg values ≥13 μg/L) among PW residing within and near the Ashkelon sub-district, which is highly reliant on DIDW [21]. However, additional research is needed in regions with different levels of reliance on DIDW to establish a fuller understanding of the relationship between desalination and ID [7, 18].

Research should also be carried out on a general community sample. Finally, it will be important to assess how reliance on DIDW affects the prevalence of neonatal hypothyroidism and the extent to which ICS intake as well as whether preconception initiation of ICS can reduce that prevalence. In the current study, along with relatively high Tg values, iodine intake was low compared to the recommendations of the WHO [11] and the IOM [30]. This may be due, in part, to negligible use of IS (used by only 4% of the PW) and low use of ICS (taken by 52% of the PW).

### Policy implications

Fortification and supplementation have a long tradition in public health practice [36–38]. Fortification of basic foods is the best way to prevent silent malnutrition [36, 38]. Around the world, iodine fortification has been practiced for nearly a century [36, 39]. In Israel, the MOH has debated the development of policy and the implementation of practical guidelines for decades [37], with gradual progress with partial salt iodization [40]. In 2011 the Israeli National Nutrition Committee 2020 published recommendations regarding fortification of salt with iodine [41]. In early 2017, the MOH published guidelines for the general public encouraging voluntary use of IS [13]. Importantly, it is currently unclear whether fortification alone can provide adequate iodine intake during pregnancy and lactation [39, 42, 43], and the range of appropriate iodine levels is narrow. Moreover, if a country moves ahead with a Universal Salt Iodization Program, then its ICS recommendations for PW must be carefully calibrated so that women of child bearing age and PW get the appropriate amount of iodine – neither too little nor too much [39, 43–48]. Intake of both IS and ICS, together with other iodine rich food sources, may potentially lead to excessive iodine intake [16, 30, 39, 49] 1.

We strongly suggest that the MOH consider the following stesp [36, 37, 42]:

- Adopt WHO guidelines for iodization of salt and the development of a national universal salt iodization program, in coordination with the ICS recommendations for PW [38, 39, 44–47, 50];
- Encourage cooperative industry engagement using regulation and financial incentives [43];
- Initiate a professional advisory board composed of clinicians, public policy makers and industry representatives;
- Promote ICS adherence and knowledge of dietary iodine sources by caregivers and possibly by health maintenance organizations;
- Promote awareness (among both professionals and pregnant women themselves) of the importance of adequate iodine intake during pregnancy; accelerating public health information campaign and professional education initiatives;
- Institute appropriate regulations and a monitoring program to ensure adequate and safe fortification and supplementation [37].

## Conclusions

In conclusion, the MOH policy currently falls short of achieving sufficient iodine status in Israeli PW, regardless of efforts to increase iodine intake in the public. This is true in particular regarding voluntary IS intake by the general population as well as voluntary ICS intake among PW, as demonstrated in this study. Thus, effective strategies to improve iodine status are warranted. Specifically, this study suggests an urgent need for governmental leadership to address ID among PW, and the broader issue of iodine deficiency also needs to be addressed. We recommend implementing a mandatory iodine fortification program in Israel, including a wellmonitored IS program in keeping with WHO standards, as well as actively promoting guidelines for ICS adherence by caregivers throughout the Israeli health system. In order to ensure optimal iodine intake among PW, the guidelines should consider total iodine intake from multiple sources.

## Data Availability

Please contact the lead author for data requests.

## Abbreviations

BMI: Body mass index
BUMCA: Barzilai University Medical Center in Ashkelon
DIDW: Desalinated iodine-diluted water
ICS: Iodine-containing supplements
ID: Iodine deficiency
INIS: Israel National Iodine Study
IOM: Institute of Medicine
IS: Iodized salt
PW: Pregnant women
SD: Standard deviation
sIFFQ: semi-iodine food frequency questionnaire
Tg: Thyroglobulin
TgAb: Thyroglobulin antibodies
TPOAb: Thyroid peroxidase antibodies
TSH: Thyrotropin
Ug/L: micrograms per liter
WHO: World Health Organization
μg/d: micrograms per day

## Footnotes

1 For example, daily intake of 1 teaspoon of IS with 30 mg/Kg (150 μg), 1 tablet of ICS with 220 μg, as well as a single 240 ml glass of cow milk with iodine concentration of 30 μg/100 g (~ 70 μg) in addition to 1 whole egg with iodine concentration of 40 μg/100 g (20 μg) and a 200 g Salmon fish fillet (~ 300 μg) or seaweed products with broad range of 11–6118 μg/g may exceed both the European or IOM tolerable upper limit of 600 or even 1100 μg/d (respectively).
